# Oxidative Stress in Prediabetic Young Adults

**DOI:** 10.7759/cureus.62504

**Published:** 2024-06-17

**Authors:** Irfan G Mulla, Ashish Anjankar, Shilpa A Pratinidhi, Sandip D Lambe, Sarita V Agrawal

**Affiliations:** 1 Biochemistry, Datta Meghe Institute of Higher Education and Research, Wardha, IND; 2 Biochemistry, Bharatratna Atalbihari Vajpayee Medical College, Pune, IND; 3 Biochemistry, SMT Mathurabai Bhausaheb Thorat Sevabhavi Trust Institute of Medical Sciences and Research Centre, Nashik, IND

**Keywords:** type 2 diabetic mellitus (t2dm), fasting blood glucose (fbg), homeostatic model assessment for insulin resistance (homa-ir), cardiovascular diseases (cvd), prediabetes

## Abstract

Background

Malondialdehyde (MDA) and nitric oxide (NO) are considered specific biomarkers for oxidative stress. Oxidative stress in prediabetics with an augmented potential for the onset of diabetes is at least partly responsible for the various complications of diabetes. Evidence shows that the early features of cell injury are due to transient acute elevations in blood glucose. This study aims to determine whether oxidative stress in prediabetic young adults increases the risk of developing diabetes.

Aim and objectives

We envisaged a study to determine whether the parameters representing oxidative stress are deranged in prediabetics.

Materials and methods

The study was conducted on prediabetic young individuals from 18 to 35 years, screened from the tertiary-level hospital, and a similar group of non-prediabetic young individuals identified from the same in a tertiary-level hospital in India.

Results

We observed significant elevations in prediabetics in the following oxidative stress parameters: MDA (P= <0.001), and NO (P= <0.001); indicating that these parameters were significantly higher among the prediabetics than the controls. We also observed significantly greater body weight, waist circumference, and BMI among the prediabetics than the controls.

Conclusion

Early identification and appropriate treatment of hyperglycemia in prediabetics is essential, as impairments in pancreatic beta-cell functioning and resistance to insulin are already present before the onset of type 2 diabetes mellitus (T2DM). Owing to the high potential for mortality and morbidity due to cardiovascular diseases (CVDs) as a complication of diabetes, treatment plans must be put in place early enough so that complications can be prevented. Inflammation and oxidative stress may be viewed as valuable targets to hinder the evolution of T2DM from prediabetes.

## Introduction

Prediabetes is defined as the presence of borderline high glycemic parameters, but with the absence of signs and symptoms of hyperglycemia, so that the person cannot be labeled as ‘diabetic’. It is shown to be an intermediate state that predisposes to full-blown diabetes mellitus [1]. Insulin resistance with subsequent insulin hypersecretion and impaired incretin action is considered critical in the pathophysiology of prediabetes. Nevertheless, prediabetes is also not considered benign, as it is shown to have a strong association with microvascular and macrovascular complications [2].

Current hypothesis suggests that oxidative stress in prediabetics is at least partly responsible for the various complications of diabetes [3]. The evidence shows that the early features of cell injury can be attributed to acute transient elevations in blood glucose. Alterations in the ratio of NAD+/NADH, generation of reactive oxygen species, and altered membrane potential of mitochondria have been reported in human in vitro hepatic carcinoma models exposed to high levels of glucose [3].

The incomplete reduction of oxygen during passage through the electron transport chain in mitochondria is the most likely source of free radicals (superoxide radical O_2_-). The subsequent oxidation of biomolecules such as carbohydrates and proteins is considered pathogenic in human diseases. The healthy human body normally mounts a natural defense against free radicals through antioxidants that inhibit the generation of free radicals or scavenge the already-formed free radicals. These processes could be enzymatic (e.g., glutathione peroxidase, catalases, superoxide dismutase, and paraoxonase) or nonenzymatic (e.g., uric acid, vitamins, proteins, and bilirubin). The zinc and copper-containing cytoplasmic superoxide dismutase enzyme scavenges superoxide radicals to hydrogen peroxide molecular oxygen, protecting against superoxide toxicity [4].

An altered balance of free radicals and antioxidants may exist in prediabetics, which may be one of the contributors to the progression of the disease to diabetes. Therefore, we envisaged a study to determine whether the parameters representing oxidative stress are deranged in prediabetics.

## Materials and methods

We performed an observational cross-sectional comparative analytical study in a tertiary-level Acharya Vinoba Bhave Hospital in Sawangi Meghe, India. The study was conducted on prediabetic young individuals, screened from the tertiary care center, and a similar group of non-prediabetic young individuals identified from the same tertiary care center. The study was conducted from 5th January 2023 to 10th April 2024.

After obtaining the requisite permission from the Ethics Committee of the institute (Letter No- D.M.I.H.E.R. (D.U.)/I.E.C./2023/1078), all patients attending General Medicine OPD in Acharya Vinoba Bhave Hospital who fulfilled the American Diabetes Association (ADA) criteria of prediabetes and give consent were included in the study.

Sample size

We enrolled 142 participants in each of the two groups: cases, which included prediabetics; and controls, which included normal healthy individuals.

Inclusion criteria

1) Age 18 to 35 years, 2) fasting blood glucose (FBG) levels between 100 and 125 mg/dl and HbA1c between 5.7 and 6.4 %

Exclusion criteria

1) Type 1 diabetes mellitus, 2) type 2 diabetes mellitus, 3) hepatic disease, 4) patients on insulin, thiazolidinediones, or metformin, 5) any malignancies, 6) other diseases and drugs including corticosteroids, octreotide, beta blockers, thiazide diuretics, statins, and antipsychotics that alter glucose metabolism, 7) pregnant women.

The screening questionnaire was also approved by the Ethics Committee of the institute. The recruited participants were classified into prediabetics and healthy controls based on the HbA1c and fasting blood sugar (FBS) as per ADA guidelines. Prediabetics were defined as those with HbA1c values between 5.7 and 6.4%and FBS between 100 and 125 mg/dl, and not on any antidiabetic treatment.

Measurement of anthropometric parameters

The body weights, heights, BMI, and waist circumference were recorded.

Collection of blood samples

After an overnight fast for 8 to 12 hours, 5 ml of blood was drawn from the antecubital vein from all subjects and evaluated for the levels of malondialdehyde (MDA), and nitric oxide (NO). The estimation of NO was done by the kinetic cadmium reduction method as described by Cortas and Wakid [5]; while that of MDA was done by the colorimetric method as described by Satoh [6]. Standardization of MDA was done and concentration was calculated as per the graph of optical density versus concentration.

Statistical analyses

The demographic data were descriptively reported as mean ± S.D. The parameters with quantitative data were assessed with the Student ‘t’ test after confirmation of its normality. All the statistical tests were accomplished with the SPSS software latest version 24.0.

## Results

We enrolled 142 participants in each of the two groups: cases, which included prediabetics; and controls, which included normal healthy individuals.

Demographics and anthropometric measures

Age

The mean age of cases in our study was found to be 27.74 ± 4.98 years, while that of controls was found to be 27.77 ± 5.00 years. The ages of the two groups were not found to be significantly different when compared with the ‘t’ test (p = 0.96) (Table 1).

**Table 1:** Ages in the two study arms

Group	Mean ± S.D. (years)	t score	p value
Cases	27.74 ± 4.98	˗0.05	0.96
Controls	27.77 ± 5.00		

Body Weight

The mean body weight of cases was found to be 84.84 ± 5.61 kg, while that of controls was found to be 66.62 ± 5.51 kg. The cases were found to have significantly greater body weights than the controls (p < 0.001) (Table 2).

**Table 2:** Body weights in the two study arms *indicates statistical significance

Group	Mean ± S.D. (kg)	t score	p value
Cases	84.84 ± 5.61	27.61	< 0.001*
Controls	66.62 ± 5.51		

Height

The mean height of cases was found to be 163.75 ± 4.65 cm, while that of controls was found to be 163.72 ± 4.72 cm. The heights of the two groups were not found to be significantly different (p = 0.07) (Table 3).

**Table 3:** Heights in the two study arms

Group	Mean ± S.D. (cm)	t score	p value
Cases	163.75 ± 4.65	1.85	0.07
Controls	163.72 ± 4.72		

Waist Circumference

The mean reading of waist circumference of cases was found to be 41.82 ± 2.53 cm, while that of controls was found to be 34.38 ± 1.88 cm. The cases were found to have significantly greater waist circumferences than the controls (p < 0.001) (Table 4).

**Table 4:** Waist circumferences in the two study arms *indicates statistical significance

Group	Mean ± S.D. (cm)	t score	p value
Cases	41.82 ± 2.53	28.13	< 0.001*
Controls	34.38 ± 1.88		

BMI

The mean BMI of cases was found to be 31.69 ± 2.50 kg/m^2^, while that of controls was found to be 24.84 ± 1.56 kg/m^2^. The cases were found to have significantly greater BMIs than the controls (p < 0.001) (Table 5).

**Table 5:** BMIs in the two study arms *indicates statistical significance

Group	Mean ± S.D. (kg/m^2^)	t score	p value
Cases	31.69 ± 2.50	27.70	< 0.001*
Controls	24.84 ± 1.56		

Biochemical parameters

Malondialdehyde (MDA)

The mean MDA levels of the cases were found to be 8.65 ± 1.08 µmol/L, while that of controls was found to be 3.94 ± 1.27 µmol/L. The cases were found to show significantly greater MDA levels than the controls (p < 0.001) (Table 6).

**Table 6:** Comparison of MDA levels between the cases and controls *indicates statistical significance

Group	Mean ± S.D. (µmol/L)	t score	p value
Cases	8.65 ± 1.08	33.667	< 0.001*
Controls	3.94 ± 1.27		

Nitric oxide (NO)

The mean NO levels of the cases was found to be 94.31 ± 10.88 ppb, while that of controls was found to be 62.79 ± 13.90 ppb. The cases were found to show significantly greater NO levels than the controls (p < 0.001) (Table 7).

**Table 7:** Comparison of NO levels between the cases and controls *indicates statistical significance

Group	Mean ± S.D. (ppb)	t score	p value
Cases	94.31 ± 10.88	21.279	< 0.001*
Controls	62.79 ± 13.90		

## Discussion

We observed significant elevations in the prediabetics in the following oxidative stress parameters: MDA, and NO; indicating that these parameters were significantly higher among the prediabetics than the controls. We also observed significantly greater body weight, waist circumference, and BMI among the prediabetics than the controls.

Our results support the findings previously published in the literature that suggest that the chronic nature of DM enhances the peroxidation of lipids and production of MDA, irrespective of the antioxidant activity and glycemic control [7]. Additionally, it has also been proposed that oxidative stress correlates with the phenotypes of prediabetes [8]. An increase in oxidative stress during prediabetes was observed in our study by the reduction in the total antioxidant capacity and raised MDA levels, which are corroborated by results from published studies [8-10]. Similarly, Su et al. [11] have also reported elevations in MDA in prediabetics as against those with normal glucose tolerance. Our findings agree with these, which may be linked with the increase in BMI and waist circumference in prediabetics, given that systemic oxidative stress has been documented to coexist with visceral obesity [12, 13]. Hence, it has been suggested that modification of the antioxidant status, favoring oxidative stress, might already be in place even before the development of diabetes [14]. Our results are also in consonance with other studies.

Certain other studies have correlated the presence of hyperglycemia with oxidative stress. Studies in a mouse model have shown that administration of a nicotinamide adenine dinucleotide phosphate (NADPH) oxidase inhibitor reduced oxidative stress and improved glucose metabolism [15]. The supposed mechanism connecting the two is impairment in glucose in adipocytes and myocytes, and reduction in insulin secretion that can be seen in oxidative stress [16,17].

Insulin resistance has also been observed in prediabetics and obese individuals in the Framingham Offspring Study, which has reported a positive correlation between markers of oxidative stress and insulin resistance [8]. Also, succinobucol, a molecule with antioxidant properties has shown antihyperglycemic activity in animal models [18] further hinting that modulation of oxidative stress may benefit in addressing the progression of prediabetes to diabetes.

Thus, our study showed an increase in the biochemical parameters in prediabetics when compared with healthy individuals. Hence, we propose that abnormalities in oxidative stress may be detected as early indicators of the long-term complications of diabetes mellitus.

However, there are certain limitations in our study, such as the relatively small number of patients so further studies are needed with large sample sizes and multicentric studies.

## Conclusions

Early identification and appropriate treatment of hyperglycemia in prediabetics is important, as impairments in pancreatic beta-cell functioning and resistance to insulin are already present before the onset of T2DM. Owing to the high potential for mortality and morbidity due to CVDs as a complication of diabetes, treatment plans must be put in place early enough so that complications can be prevented. As shown in our study, oxidative stress can be present early on during the course of prediabetes, which can be evidenced by derangements of the corresponding biochemical parameters. Further studies are needed which can attempt to manipulate the oxidants and antioxidants in the human body, toward antioxidants, to inhibit the inflammatory processes that are central to the pathophysiology and progression of prediabetes to diabetes.
